# Judging the credibility of websites: an effectiveness trial of the spacing effect in the elementary classroom

**DOI:** 10.1186/s41235-022-00358-w

**Published:** 2022-01-17

**Authors:** Vanessa Foot-Seymour, Melody Wiseheart

**Affiliations:** 1 Department of Psychology, York University, Toronto, Canada; 2 LaMarsh Centre for Child and Youth Research, York University, Toronto, Canada; 3 York Region District School Board, Aurora, ON Canada

**Keywords:** Spacing effect, Classroom, Critical thinking, Credibility, Higher-order thinking

## Abstract

Spaced learning—the spacing effect—is a cognitive phenomenon whereby memory for to-be-learned material is better when a fixed amount of study time is spread across multiple learning sessions instead of crammed into a more condensed time period. The spacing effect has been shown to be effective across a wide range of ages and learning materials, but few studies have been conducted that look at whether spacing can be effective in real-world classrooms, using real curriculum content, with real teachers leading the intervention. In the current study, lesson plans for teaching website credibility were distributed to homeroom elementary teachers with specific instructions on how to manipulate the timing of the lessons for either a one-per-day or one-per-week delivery. One month after the final lesson, students were asked to apply their knowledge on a final test, where they evaluated two new websites. Results were mixed, suggesting that classroom noise might lessen or impede researchers’ ability to find spacing effects in naturalistic settings.

**Supplementary Information:**

The online version contains supplementary material available at 10.1186/s41235-022-00358-w.

## Introduction

The goal of the learner is seemingly simple—they strive to acquire new knowledge so that they can understand, retain, and retrieve it when needed. Classrooms are full of students who are trying to do just that. But each year, they remember some concepts and forget others. Teachers are given the task of figuring out which students remember what, how well they remember it, whether they can apply it, and how much they will need to re-teach it to bring students back up to mastery before adding on new knowledge that builds on existing content. This can be a daunting task for any teacher. In order to ensure that students are on track with their learning goals, teachers are required to follow current curriculum guidelines, which include expectations and outcomes for their students during and at the end of each school year. Although teaching the content listed in these guidelines remains a central task for teachers, there are still unanswered questions about how to implement the content to enhance student retention.

When designing a curriculum document, there are many aspects of learning that need to be addressed. Content is of the utmost importance, as are the aims and objectives for learning, assessment, and educational strategies for implementation (such as problem-based learning). Roles for parents, teachers and students are discussed, as are best practices for delivering the many expectations that are listed as both specific and overall goals. However, a review of the most current curriculum documents in Ontario (Ontario Ministry of Education, 2006) demonstrates that although the curriculum provides some further guidance and information to support its implementation (especially when it comes to modifying and accommodating programming for students), there is no direct connection to strategies from the field of cognitive science which may help to boost retention (those that have been recommended by the National Council on Teacher Quality, 2016).

The NCTQ (National Council on Teacher Quality) is an American research and policy group that was founded by the Thomas B. Fordham Institute, who serve to overhaul education and challenge its current system. One of the documents produced by the NCTQ recommended that in order to boost student retention, teachers should use any and all of the following strategies: pairing graphics with words; linking abstract concepts with concrete representations; posing probing questions; interleaving problems; assessing students; and distributing practice (i.e., spacing out review sessions). Their recent review of hundreds of relevant teacher education textbooks demonstrated that almost 60% of these texts fail to mention even one of these six fundamental instructional strategies, and those who do fall short in explaining that strategy properly (NCTQ, 2016). The NCTQ’s review also found that adjusting timing of lessons via practice to boost retention is still a relatively unexplored and underutilized area (Harden, 1999). Practice is a standard part of most teachers’ lesson plans, seen most commonly in homework review and daily activities, but practice is not the only required piece of the puzzle. Depending on the interval between instruction and practice, the timing of practice can have massively different effects on student learning and retention.

Thankfully, if teachers want to adjust timing of practice to boost retention, there is an abundance of scientific data on how to implement a successful distributed practice intervention (Wiseheart et al., 2019). However, a barrier to wide-scale implementation is that there is insufficient evidence to show whether or not systematically modifying lesson timing is worth the extra effort that it may entail. Therefore, the focus of this paper is to (1) summarize the existing literature on the spacing effect, (2) point out the gaps in the existing literature that may be the reason for the delay in school implementation, and (3) fill those gaps with a new, large scale effectiveness study on lesson timing in the classroom, using both fact and critical thinking materials.

### Spacing effect

In the psychology literature, the spacing effect (also called distributed practice), refers to the boost in retention that occurs after newly learned information is relearned or restudied across multiple smaller chunks of time, as opposed to learned once in a longer chuck of time. Given equal amounts of time spent studying, spacing has been shown to boost long-term memory (Cepeda et al., 2008, 2009).

In a typical spacing paradigm, the learner is given new information to memorize (e.g., a list of words). Learners are often separated into two groups: massed and spaced. The massed learner spends some time learning the information and then reviews (practices) the same information in repetitive blocks (occurring one immediately after the other with little or no time in between). The spaced learner is given the same information to learn but instead of having the blocks appear one immediately after the other, they are given some time in between the blocks before restudying (this is called the inter-study interval)*.* In the literature, these are also called practice or review sessions, because in order to use spacing effectively, repetition of the same items is key. After a fixed amount of time following the final learning episode has passed (called the retention interval), students are tested on the information to see how much they remember.

### Spacing benefits to learning

Many studies, reviews and meta-analyses have been conducted on the spacing effect (Cepeda et al., 2006; Delaney et al., 2010; Dempster, 1996; Janiszewski et al., 2003; Kupper-Tetzel et al., 2014; Maddox, 2016; Toppino & Gerbier, 2014; Wiseheart et al., 2019). These papers explain that spacing has been shown to improve memory for many different types of content, such as basic vocabulary (Bloom & Shuell, 1981), random facts (DeRemer & D’Agostino, 1974), textbook concepts (Reder & Anderson, 1982), word lists (Zechmeister & Shaughnessy, 1980), addition (Reed, 1924), multiplication (Rea & Modigliani, 1985) and geometry (Rohrer, 2009; Rohrer & Taylor, 2007; Taraban et al., 2001). Spacing effects have been seen across age groups. Benefits have been seen in infants (Rovee-Collier et al., 1995), elementary and middle school children (Carpenter et al., 2009; Foot-Seymour et al., 2019; Sobel et al., 2011), high school students (Bloom & Shuell, 1981; Küpper-Tetzel et al., 2014), and healthy adults, including older adults (Cepeda et al., 2008; Simone et al., 2013).

Reported effect sizes for spacing in the verbal (fact-learning) literature are the largest (*d* = 0.85: Cepeda et al., 2006; Moss, 1995), and accumulating evidence suggests that the magnitude of spacing effects may depend on type of content or the skill that is being learned (Wiseheart et al., 2019). For example, the estimated effect size for non-verbal realms is predicted to be a bit lower (*d* = 0.5), based on the studies that have been conducted using this type of material (Foot-Seymour et al., 2019; Gluckman et al., 2014; Kapler et al., 2015; Vlach & Sandhofer, 2012).

### Spacing in the classroom

Spacing studies have traditionally focused on teaching rote memorization in controlled laboratory settings, which is extremely different from the wide array of learning that takes place in the classroom. As well, there aren’t yet enough classroom studies to support use of the spacing effect across the entire range of educational materials. Some of the applied classroom-based studies that have been conducted with verbal and factual material show spacing benefits for word and phonics learning (Seabrook et al., 2005), word and fact learning (Carpenter et al., 2009; Sobel et al., 2011), second language learning (Bloom & Shuell, 1981; Küpper-Tetzel et al., 2014), and text comprehension (Rawson & Kintsch, 2005; Verkoeijen et al., 2008). These studies all showed benefits of spacing.

Previous classroom studies have shed some light on the difficulties of planning and conducting real-world classroom research, and why this type of research might be so scarce in the literature. In a spacing study by Foot-Seymour et al. (2019), there was a delay in the starting of the research due to a school board-wide strike, and when classroom learning began, lessons were interrupted by several snow days, fire drills, and alternate class programming. The experimental noise of the classroom setting might be an intimidating place for researchers to venture, especially since researchers are used to conducting studies with more rigorous control.

Even if classroom studies were conducted more often, there could be criticism that students need to start going beyond simple fact learning to integrate critical thinking (i.e., higher-order thinking) skills. Rote memorization and fact learning has its place in the classroom, of course—student success is heavily dependent on a foundational knowledge base in every subject. The problem arises when students are asked to go beyond the basic facts and apply them in problem solving situations where they need to interpret, analyze, evaluate, explain, and make inferences. These critical thinking skills are core to many disciplines (and in many workplaces).

### Spacing and critical thinking

**Fig. 1:**
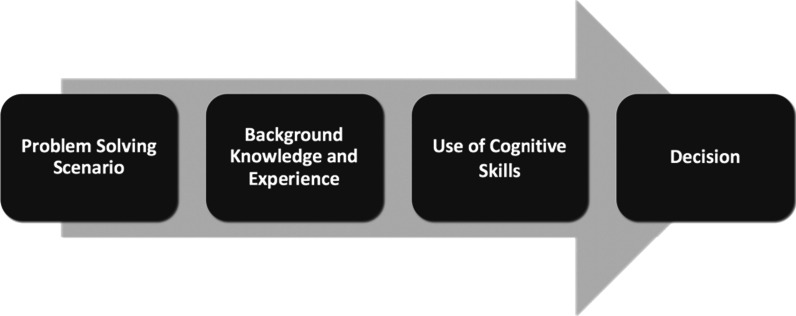
A summary of the critical thinking process by Ennis (1987, 2018)

Robert Ennis (1987, 2018) defines critical thinking as “reasonable, reflective thinking that is focused on what to believe or do” (p. 10). Other definitions of critical thinking exist (Facione, 1990; Kuhn, 1999; Siegel, 1988), but Ennis describes them as smaller pieces of a larger conceptual pie. A key commonality is that critical thinking is goal-oriented—a good critical thinker evaluates their options before coming to a well-reasoned decision. Critical thinking is also most effective when the individual has some background knowledge and experience in the field in order to be able to engage in the full process (Ennis, 2018; Fig. 1).

**Fig. 2:**
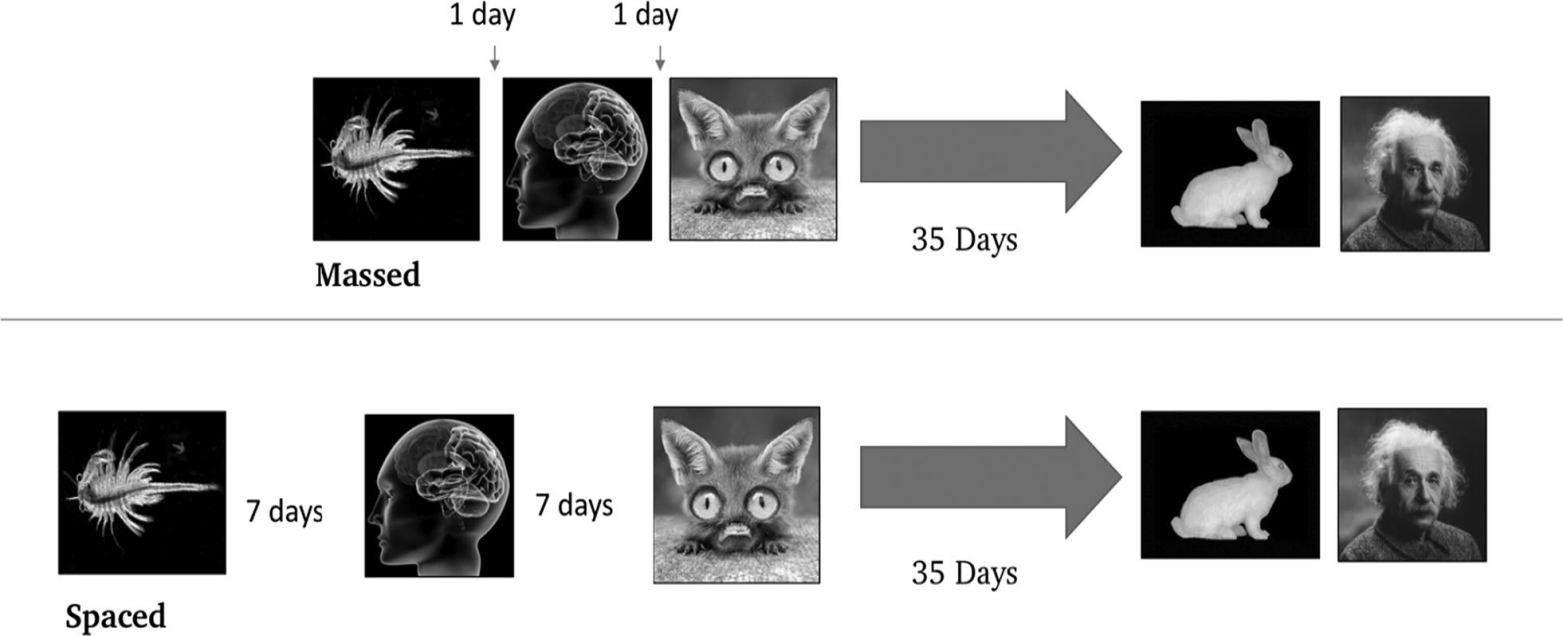
Visual representation of the lesson timing for the current study (massed, spaced). Each photograph represents a new website where students practiced their website evaluation skills

Critical thinking is an important tool if we are to maintain our roles within a democratic society (Dewey, 1909). As such, breaking down its components is necessary so that we can train our newest generation of thinkers. As citizens, students need to obtain a critical view of the world instead of simply accepting the thoughts and opinions that are placed upon them—especially now that they are being constantly exposed to information not only through school, but also at home via the Internet. Given that critical thinking is at the forefront of many education policy documents (Fullan, 2013) and is of vital importance to student learning, it is surprising that it has largely been ignored by learning and memory researchers. To date, less than 1% of spacing effect research studies have examined the learning of critical thinking skills.

In the literature, critical thinking is sometimes referred to as higher level, or higher order thinking because of its connection to a pre-existing framework called Bloom’s Taxonomy (Bloom, 1956). Bloom’s Taxonomy is typically represented as a pyramid where knowledge is at the bottom (low-level thinking), followed by comprehension, application, analysis, synthesis and evaluation. Although convenient, it is not entirely appropriate to categorize thinking in this way, since thinking is not hierarchical in nature. Instead, the ability to recall and retrieve information reinforces knowledge, which then provides the backdrop for all further learning.

There have only been a few studies that have looked at spacing and critical thinking content in the classroom, all with varying definitions of critical thinking. Butler et al. (2015) implemented three cognitive science principles, including spacing, in the university classroom using curriculum-based materials. This study used higher order thinking questions, asking students to solve STEM problems in either one session (massed) or across three weekly sessions (spaced). There were several different retention intervals (two to four weeks), and Butler et al. found that any spacing was better than no spacing—the materials that had been practiced one week later were retained better on the final exam.

Another study looked at spacing in a simulated undergraduate classroom and investigated long-term benefits for factual and higher-level learning, as classified by Bloom’s Taxonomy (Kapler et al., 2015). The researchers hosted a university lecture where they presented students with science material. Students were asked to review the material after either one (massed) or eight days (spaced), and five weeks after the last lesson, students completed a final test. Final test questions consisted of either factual or higher-level application questions. Reviewing the material in the spaced condition was more beneficial for both factual and higher-level questions on the final test.

Vlach and Sandhofer (2012) looked at spacing and critical thinking (defined as an ability to make generalizations) in students aged 5–7 years. Researchers taught and tested students in their university laboratory school. The researchers asked students to study facts about food chains and then tested their ability to generalize about the consequences of what happens when that food chain is disrupted. Students were in one of three conditions: massed study sessions where all learning and reviewing occurred in one day; spaced study sessions that were spread across two days (which they referred to as clumped); or spaced study sessions that were spread across four days. Children were tested after a one-week retention interval. Students who were in the spacing conditions showed retention benefits for both the factual materials and the ability to generalize from what they had learned. Gluckman et al. (2014) replicated this study but added a memory component that added more fact learning content in addition to the generalization content. Memory for facts surrounding food chains was significantly better in the spaced conditions than in the massed conditions, and as expected, there also was a spacing advantage for both simple and complex generalizations of concepts.

Foot-Seymour et al. (2019) is the most recently published spacing and critical thinking study. This study defined critical thinking as goal-oriented and directional, where a sturdy knowledge base is built and then decisions are made before coming to a goal. The current study defines critical thinking in the same way. This particular study of the spacing effect was conducted in actual classrooms, where researchers implemented and taught a critical thinking curriculum unit on website evaluation, which was based off of the standard media literacy curriculum. The same teacher/researcher taught all of the lessons to students. A total of 558 students in grades 4–6 (9–12 years old) were randomly assigned to either a massed condition (three days in a row), or spaced condition (three lessons one week apart). As expected, students who took part in the weekly lessons had a statistically significant spacing advantage on the final test 35-days later for both the fact and critical thinking measures. Specifically, students in the spaced condition remembered more from the website credibility lessons and were better able to explain their website ratings than students in the massed group. Since the current study is based on this previous research, Table 1 highlights the differences between the studies. The most important thing to note is that the current study is a replication, with changes made to decrease researcher intervention and increase teacher participation. Revisions were also made to some of the materials (websites were made by the researchers; the checklist was revised to make it shorter).

**Table 1:** Methodological differences between Foot-Seymour et al. (2019) and the current study

	Foot-Seymour et al. (2019)	Current study
Lesson delivery	Researcher taught students in person and facilitated all aspects of the lessons	Researcher pre-recorded videos and classroom teachers facilitated all aspects of all lessons with the exception of the first lesson
Materials	All materials were on paper	All materials were online
	Pre-existing websites were used	Websites were created by researchers
	Checklist (17 questions)	Revised checklist (14 questions)
Final test	Students evaluated one website at final test	Students evaluated two websites at final test

Researchers taught lesson one in order to teach the teacher how to run the lessons

### Critical thinking and website evaluation

According to Ennis, critical thinking involves a set of pertinent skills and dispositions that should be taught explicitly and infused to everyday life in order to create an implicit understanding. These skills and definitions are listed in the Alpha Conception Report (Facione, 1990)—a report outlining a list of six cognitive, or critical thinking skills: interpretation, analysis, inference, evaluation, self-regulation, and explanation. Furthermore, critical thinking skills are best applied when embedded in a subject context. In a meta-analysis of critical thinking skills in the classroom, Abrami et al. (2008) found that instruction of critical thinking was most effective when students were taught critical thinking instruction and subject content in approximately equal parts. This led to their recommendation that teachers should teach critical thinking skills so that students are able to put them into context and learn to use them before transferring them to other disciplines. Students should be given practical and relevant examples of when they might use their developing critical thinking skills, such as website evaluation. In line with this approach are the findings from Facione (1990) and other research suggesting that subject matter should be taught with critical thinking skills training, as opposed to the latter taught separately (Angeli & Valanides, 2009; Ennis, 2018; Facione, 1990).

### Current study

If spacing is to be used in the classroom without researcher support, more evidence is needed to see if teachers can lead the intervention on their own using traditional lesson plans and minimal instruction. Teachers were provided with lesson plans and research timing parameters, including the specific dates when material should be taught, reviewed, and tested. Teachers executed these lessons during their usual literacy block, since lessons were embedded with curriculum-based content and taught by the participants’ homeroom teacher. Students participated in the same three lessons whether they were in the spaced learning condition (weekly lessons: 7-day inter-study interval [ISI]), or the massed condition (daily lessons: 1-day inter-study interval [ISI]). All students were given a final test approximately 35 days later. This was chosen as the retention interval because it is the optimal retention interval (RI) for a 7-day spacing condition (Cepeda et al., 2008), and because it was the ISI and RI combination in a closely related spacing effect study (Foot-Seymour et al., 2019). Additionally, it is feasible for a teacher to plan their lessons with a one-week spacing design with a recommended testing date of one month from the last lesson. Since spacing benefits are present across a very wide range of retention intervals, the same results should be obtained even with a different ISI and RI combination (Cepeda et al., 2006, 2008).

### Hypotheses

The hypotheses were as follows: First, the spacing effect will benefit fact learning. Students in the spacing condition, when cued, will recall more information from the lessons (the four categories of website evaluation: design, authority, content and purpose; Table 2) than students in the massed condition. This was prompted by asking students, “What are the four categories of website evaluation?” Second, the spacing effect will benefit critical thinking: Students in the spaced condition will spontaneously use more critical thinking criteria and website details in an open-ended paragraph, by giving details taught in the lessons to explain their website ratings, than students in the massed condition. This was prompted by giving students two different websites and asking for each, “Is this website credible? Please explain using evidence from the website.”

**Table 2:** Website evaluation checklist. Adapted from Bronstein (2007) and Foot-Seymour et al. (2019)

**Design**	
Do the photos and colour choices look professional?	
Is the website nicely organized and easy to navigate?	
Are there any obvious spelling errors or typos?	
Is the layout consistent from page to page?	
**Authority**	
Is the author/creator of the website clearly identified?	
Is the author of the website an expert in their field?	
Is there a way to contact the author by phone, mail or e-mail?	
**Content**	
Does the website say when it was created?	
Does the website say when it was last updated?	
Can you confirm that the information is correct by doing a Google search?	
Are the links relevant to the subject? In other words, do the links take you somewhere that makes sense if you click on them?	
**Purpose**	
Is the website trying to educate you with real information?	
Is the author trying to sell you something?	
Do you think the author has intentionally left out any important information that could help you decide if it’s real or fake?	

Websites were designed by the lead researcher and student volunteers with a rating in mind. Following the above checklist, each of the five websites (three during the lessons and two during the final test) that students were asked to rate from 0 to 10 had a specific answer (e.g., the pre-test website should have been rated a 5 since 50% or 7 out of 14 of the answers to the website evaluation questions were yes and the other half were no).

There were additional hypotheses tied to the ratings, which reflected traditional spacing effect paradigms—specifically, the idea that spacing creates a “desirable difficulty” (Bjork & Bjork, 2011), where difficulty is challenging in the moment, but beneficial to memory in the long term. In order for this study to be compared to previous spacing studies, we tested a third and fourth hypothesis. Our third hypothesis is that students participating in the daily lessons will be better at rating websites during the daily lessons than students who had weekly lessons. Specifically, the massed group will stay closer to the correct rating (represented by a smaller difference score) than the spaced group. Fourth, at final test, the spaced group will rate the websites more accurately than the massed group.

In order to address these hypotheses, we used a typical spacing paradigm. Students participated in three study sessions (lessons) covering the same conceptual information, separated by an inter-study interval. The three lessons were designed to mimic standard teaching practice. After a 35-day retention interval, students completed a final test assessing their retention and ability to use the information from the lessons (Additional file 1).

## Method

### Participants

Elementary school students from York Region District School Board, aged 10–14 years, participated in this study. This age group was chosen because the Ontario curriculum states that, starting at approximately 10 years old, students must begin to value critical literacy and “differentiate between fact and opinion; evaluate the credibility of sources, and recognize bias” (Ontario Ministry of Education, 2006, p. 13). There is no formal curriculum material that asks students to draw upon their critical thinking skills as we have described in this paper, and none that asks them to evaluate websites. It is the responsibility of the teacher to implement this training in their program, and implementation varies widely between teachers. In order to randomize teacher effects, we focused on recruiting a large sample size with an even distribution of student ages across both conditions.

A total of 1054 students were recruited for the website credibility lessons, from 16 participating schools across York Region District School Board. There were 42 participating classrooms, each with its own homeroom teacher. Of the students who were recruited, three did not receive parental consent to participate and parents asked that their child be given alternative programming during the lessons. One full classroom was excluded from the final data set due to a teacher-altered spacing schedule (this classroom used a spacing schedule of 4–5–4 days instead of the requested 7–7–7 days). A further 36 students were given parental permission to participate in the lessons but asked researchers not to use their data for the analyses. Since there were four lessons, including the final test, and all were necessary to collect a full data set from each student, a total of 160 students were excluded from data analyses due to missing a lesson (e.g., due to missing a day at school for illness or school activity). Since teachers were encouraged to include all students, including those who were on an individualized education plan, who typically would have been removed from class and placed into their special education resource teacher room, it is possible that some of these students left for their regular programming on at least one of the days, which would have created some missing data. Some students (*n* = 15) were excluded from the analysis because they were English language learners and did not read and write English without full support—however, English language learners were given the help they needed via a one-on-one teacher or Google Translate so that they could still participate in the lessons as much as possible. All efforts were made to ensure that our research practices were fair and equitable. The final sample consisted of 716 students (*n* = 349 spaced; *n* = 367 massed), with a mean age of 11.8 years old (SD = 1.1) for the spaced group and 12.0 years old (SD = 1.1) for the massed group. For a more comprehensive overview of participants from the final sample, see Table 3.

**Table 3:** Overview of final sample

	Spaced (*n* = 349)	Massed (*n* = 367)	Overall (*n* = 716)
Gender identity*			
Male	175	197	372
Female	165	169	334
Other	1	0	1
Prefer not to answer	8	1	9
Grade			
5	56	39	95
6	125	101	226
7	85	122	207
8	83	105	188
Age (years)			
10	41	31	72
11	122	94	216
12	87	120	207
13	74	98	172
14	25	24	49

*As reported by participants

Our sample size surpassed our minimum recruitment aim, which was *n* = 114 per group at analysis. We based sample size on an effect size of *d* = 0.48 and 95% power, our estimate of the effect size for critical thinking and spacing based on the most related prior classroom study (Foot-Seymour et al., 2019). We aimed for a much larger sample to account for differences in teacher effectiveness, aiming for a sufficient sample size that mean teacher effectiveness would be approximately equivalent across groups. Since we do not know the distribution of teacher effectiveness, and thus cannot determine the minimum required number of classrooms, we aimed to recruit as many classrooms as possible during the time available for data collection. Given our large sample size, our power to detect an effect was 99.99%.

Since it is not standard procedure within schools to collect equity and identity-based data from students due to ethical considerations, census data for York Region were reported instead. Demographic census data demonstrate that 51% of York Region’s population are Caucasian and 49% are from a visible minority. Out of those identifying as a visible minority, 45% self-identified as Chinese, 22% as South Asian, 8% as West Asian, 5% as Black, 5% as Filipino, 3% as Korean, 3% as Southeast Asian, 3% as Latin American, 2% as Arab, 1% as Japanese, and 4% as multiple or another visible minority. More details on York Region demographics are available on the Public Tableau website (Regional Municipality of York, 2016).

### Design

A between-subjects design was used, where classes were randomly assigned to either the spaced or massed condition, stratified to ensure that there were an equal number of grades and locations for each condition. The massed condition was used as a control. Students in both conditions were given an identical set of lessons but received the lessons daily (massed: three days in a row) or weekly (the same day of the week for three weeks). Although traditional verbal learning (rote memorization) spacing effect studies have only used two study sessions (Cepeda et al., 2006), three study sessions (lessons) were taught to mimic standard teaching practice, and to repeat the methodology used in Foot-Seymour et al. (2019), where a third lesson was added so that students could experience more variability of websites and have an additional chance to review the content.

Classes were taught on each day of the week, with specific days varied across classrooms, and there was a mixture of days in each condition. Volunteers were sent to classrooms on each day of the study to assist with students who needed extra support, and to ensure that teachers were carrying out the intervention in the agreed upon schedule. The volunteers’ presence was non-intrusive and did not affect the teacher’s ability to implement the lessons as per their usual teaching practice.

Materials were introduced to students online, with brief, pre-recorded lessons. After the videos were complete, students went on a self-led exploration of the website, completed the corresponding checklist, and shared their findings with the teacher via a discussion. Teaching course material by showing videos is standard teaching practice—teachers were responsible for circulating, managing student behavior, answering questions, and leading the discussion.

### Materials

#### Websites

All websites were created by the researchers on WordPress. Each was designed to have a specific level of credibility (3, 5, or 7 out of 10), with at least one of the four categories scoring very low (Table 4). Red flags (deliberate errors) were embedded throughout the websites to encourage a scavenger-hunt feel while students were going through the checklist. We used dedicated non-Wordpress domain names transparently linked to a paid Wordpress website during the study. We provide links to permanent unpaid Wordpress websites in the manuscript.

**Table 4:** Websites used during credibility lessons

Website	Rating (/10)	Design (/4)	Authority (/3)	Content (/4)	Purpose (/3)	Total (/14)
Sea monkey online	5	3	0	1	3	7
Brain science	3	4	0	0	0	4
Bizarre animals	7	4	0	4	2	10
Glowing bunnies	7	1	3	3	3	10
Association of geniuses	3	2	0	1	1	4

Rating values represent correct rating out of 10, and individual category numbers show which categories scored high/low to lead students to that decision

#### Sea Monkey Online (seamonkeyonline.wordpress.com)

**.** “*This website is all about the marvellous creature, the sea monkey! Feel free to browse, search and comment*.” This website taught students about the true history of the sea monkey, in an error-ridden blog formatted website. There were distracting spelling errors all over the website. The information provided was true, but the authorship was up for debate. The website claimed to be written by “The Office of Science and Society” at “MacGill University” but gave no author name or credentials, and the hyperlink that was connected to that name took students to a different website run by the real McGill University. The author name was still embedded deep within the McGill University website. Some students noticed the error in the university name on the Sea Monkey website immediately and others did not, but regardless, all students were taught that they needed to pay attention to small details and trust their gut when it came to making decisions about specific website items to see if they were red flags (errors) or not.

#### Brain Science (brainsciencenow.wordpress.com)

This website was based on the myth that people only use 10% of their brains and stated there was an expensive pill that could change that. The purpose of the website was in question, asking for large sums of money in exchange for this super pill. “*Neuroflex is the first ever pill that allows humans to improve their brain power! It allows you to activate more regions of your brain and guarantees obtaining the maximum results with the minimum amount of effort. Neuroflex consists of a few essential ingredients that are important in enhancing brain function. It is 100% natural, with all ingredients extracted from plant sources.*” One of the defining features of this website was that it was visually pleasing and very professional looking. There was an author name on the website (Dr. Daniel Reid), and the site header gave credit to the “International Journal of Brain Science.” It also appeared to have very scientific-sounding information. However, this was the least credible website of the lessons, identified by the false information that students would have noticed while doing a Google search of the content. Also, most students noticed that the website was trying to sell them a very expensive pill (in the currency of British pounds), which was sold in a bottle that, unlike the rest of the website, did not look professional.

#### Bizarre Animals (bizarreanimalworld.wordpress.com)

This website took students through several strange animals, like the giant squid. It taught real content about seemingly bizarre creatures. “*The giant squid lives in the depths of the ocean. Giant squids can grow to a tremendous size due to deep-sea gigantism. Recent estimates put the maximum size at 13 m for females and 10 m or males from the back fins to the tip of the two long tentacles (second only to the colossal squid) at an estimated 14 m (46 ft), one of the largest living organisms.*” Inspiration from this website’s content came from the story of the Gulper Eel, a rare deep-sea creature that can stretch its jaws in a remarkable way. Other animals were added to the website that were equally unusual. They were so rare that they would have needed to check their credibility before claiming certainty. There were also red flags in the author category (each post lists the author as “staff”), which at this point the students would have known and needed to review in order to successfully rate the website.

#### Glow-in-the-Dark Bunnies (researchsciencetoday.wordpress.com)

“*In normal light, these rabbits all look normal—cute, fluffy, and white. But wait until you turn off the lights. The rabbits glow fluorescent green!*” This website shared real research about glow-in-the-dark animals, but the design looked unofficial, with a lime green background, blurry photos, and red text. Many students noticed that although the information on the website was true, there was not much content listed that could help them make their decision. Most of the content listed were hyperlinks that brought them to other sites. There was also a red flag in the form of a picture of a regular rabbit, with the heading, “to compare, here is a photograph of a normal rabbit.” Many students noticed that this photograph was out of place on this scientific-seeming website.

#### Association of Geniuses (associationofgeniuses.wordpress.com)

“*Sharing biographies of geniuses around the world.*” This website was part Albert Einstein biography and part advertisement for an association that supposedly aimed to help young geniuses discover their full genius potential. The purpose of this website was twofold, and the content was completely false. This website had a very simple design but had inconsistent fonts on every page. Students were told that they could donate to provide an hour of tutoring for a child, but until that point the website was not convincing enough to give them confidence that this would have been a good idea (as noted from the website responses students gave during this lesson). The donation button was not connected to any sort of account, so if students tried to see whether they could have donated to the cause, they would not have been able to.

#### Website credibility checklist

The website credibility checklist (Table 2) was originally adapted from Bronstein (2007) for use in the prior website credibility and spacing effect study (Foot-Seymour et al., 2019). Bronstein created a credibility checklist with the assistance of a Delphi panel of experts and explored the reliability and validity of this checklist for classroom use. She summarized a variety of commonly used checklists and designed her own based on a mixture of best practices by other educators. The checklist was designed so that students could have little to no background knowledge or critical thinking vocabulary and could be encouraged to respond with more than a simple “yes” or “no” assessment while proceeding through the list. She argued that instead of checklists with only binary options, continuous scales should be used, since critical evaluation is an ambiguous process that involves many different options for premises and different forms of reasoning that are equally legitimate. The goal was to gain deeper insight into students’ thought processes. Instead, students would look at the category (design, authority, content, and purpose) and write a response to explain their thinking. The full checklist was finalized by the Delphi panel and aimed for delivery to high school students.

Students were asked to make a final decision about their overall evaluation of the website, but their final decision was turned into a continuous scale so that students could give a value from 0 to 10 (0 being least credible and 10 being most credible) and explain this rating in a paragraph. Ideally, students would use a combination of these tools (the checklist, rating scale, and written paragraph) in order to formulate an opinion. The paragraph and subsequent final rating of the websites was a very important step in the credibility process, since the paragraph explaining the rating was intended to justify why the student gave the answer that they did.

The checklists appeared to work as expected. After going through the checklists, students were successful in matching their rating with their explanation. Even on the pre-test, before the lessons were taught, students seemed to be pairing their answer with their rating—like this student, for example, who rated the Sea Monkey website a 5/10 during the pre-test: “I do not think that this website is that credible overall. You can learn some information from it that seems true but, on the other hand some of there [sic] information is not very believable. The credibility of this website in my opinion is in the middle.” This student had not been introduced to the checklist where they learned to explain their rating. Still, they were able to successfully communicate that although they could formulate an answer (of not credible overall), they were still going back and forth. Other students struggled with their written answers in the pre-test, explaining that “I think that the website is pretty bad and I don't trust it. I don't know why. I wish you could just tell me what to do because I don't know what to do. I went through the website and read it but I can't tell.”

After the lessons had begun and students were taught to explain ratings using evidence from the website, answers became much more substantial, and still matched their ratings. On the Brain Science website during the second lesson, one student gave the website a 3/10, correctly identifying that:

The content was not too terrible at first, but they included a myth. It included some educational facts about the brain. The author might've been made up. According to a google search, the author isn't even a neuroscientist, what he said he was. The design looked professional, but I don't think the rest was. I feel that the main purpose of the website is to sell NeuroFlex, something that they say will increase how much you are able to use your brain power. I think that is ridiculous, considering the fact that they said you have to take 18 pills a day; 1 pill per hour. It included reviews, and it included a name of an author of a review. I looked up that name and it was spelt wrong but it was from the right place though. All of that made me think that this website is not really credible.

### Procedure

After university ethics approval and school board permissions were obtained, schools and classrooms were contacted in person and via e-mail and selected based on principal and teacher interest. When a teacher agreed to participate and the principal gave approval, lesson plans were sent (available at https://osf.io/9zjt2/), and dates were pre-selected by the researcher. Teachers were required to participate in the condition that they were assigned to. Communication with teachers was frequent to ensure that all aspects of the research were going smoothly, and a researcher or volunteer was physically present for every teaching session. Consent forms were collected by teachers before the lessons began. Consent forms had three options: (1) students could participate in all aspects of the lessons and have their work used anonymously for research purposes; (2) students could participate in all aspects of the lessons but could not have their work used anonymously for research purposes (responses could be recorded initially but needed to be deleted before the analysis); and (3) students could not participate in any aspect of the lessons. If parents chose the third option and did not consent, they were contacted by the teacher to confirm and ask whether students could do alternative programming in the room or if parents would like to send them to another classroom. Only three parents chose this option (*n* = 3); these students were removed and placed in another class to complete a task assigned by their homeroom teacher. The 36 students who did not receive consent to have their work used for research purposes (*n* = 36) participated in all aspects of the lessons (in-person and online), but when the survey prompted them to give their identification, they were told to mark the box with an “X” so that their responses could be deleted from the system. Consent forms were collected to ensure that these students did not have their responses recorded and saved. In-class consent management was done quietly and efficiently so that other students were not aware of who was in each consent condition, in an attempt to keep classrooms inclusive and equitable (Additional file 1).

All lessons were online, with videos and questions programmed on Qualtrics. On day one, the lead researcher (a certified teacher in Ontario) was present to meet the students and teacher and show them how to access the survey via URL. Headphones were provided to students who did not bring them from home. Students were given instructions on how to access the online lesson. Teachers were encouraged to ask questions about the next several days of learning while the researcher was present. See Table 5 for an overview of what happened during each lesson, and Fig. 2 for a visual representation of the lesson timing.

**Table 5:** Overview of lesson delivery: each lesson represents a day of the intervention

Pre-test and lesson one	Lesson two	Lesson three	Final test
Students evaluated a website before learning any content Students were taught to use the checklist with the same website as in the pre-test Students participated in a teacher-led discussion about the website	Students briefly reviewed the content via pre-recorded video and practiced evaluation with a new website Students participated in a teacher-led discussion about the website	Students briefly reviewed the content via pre-recorded video and practiced evaluation with a new website Students participated in a teacher-led discussion about the website	Students evaluated two new websites

A total of 80–100 min was allocated per class for the first day. At the beginning of the lesson, students watched a short introduction video which contained the definition of credibility and completed a pre-test website evaluation of the Sea Monkey website. For this evaluation, students were asked to explain why they thought the website was credible or not, prior to taking part in the lessons. Responses were required to have a minimum of 150 characters (approximately three sentences). The pre-test measured student responses at baseline.

After the pre-test, students watched a YouTube video (pre-recorded by the researchers) which led them through the credibility checklist using an example from a National Geographic website. After the video finished, they were asked to go through the checklist for the Sea Monkey website again, give another rating out of 10 now that they could make a more informed decision, and explain their rating using the four categories (design, authority, content, and purpose) in a paragraph. After about an hour, classroom teachers led students in a group discussion, where students could share their answers and highlight all of the red flags that students saw on the websites. The first discussion showed that students were already thinking critically—they were engaged in discourse about the categories of website evaluation (e.g., for the design category, some students commented that “the website looks like a blog so it can’t be credible,” while others said, “it had good contrast with the white background and black font. It could have been worse”). The discussion was held for approximately 10–15 min, or until students were finished sharing their ideas. Responses were recorded on the board in point form but were later erased to prevent students from any additional studying.

Lesson two was led by the homeroom teacher either one day or one week later. Students were randomly placed into small groups and were asked to brainstorm the four categories and 14 questions that they had previously learned, as retrieval practice. Responses were recorded on a chart paper that was later destroyed to prevent studying. Then, students went on the second website (Brain Science) and used the online website evaluation survey to record their responses. Lastly, students verbally shared their answer as a class in a group discussion format as they had done in the first lesson.

Lesson three was identical to lesson two, but with a new website (Bizarre Animals). Students completed the small group retrieval practice activity, then the online website evaluation survey, and then had their class discussion.

For the final test, which took place 35 days after lesson three, students were asked to complete three tasks. First, they were asked to recall the four categories of website evaluation (design, authority, content, and purpose). Next, they were given two websites (Glow-in-the-Dark Bunnies; Association of Geniuses) and asked to evaluate them one at a time, give them a rating out of 10, and write a paragraph (without the checklist) supporting their rating. This was an identical format to what students completed during the pre-test. Written responses were required to have a minimum of 300 characters. In order to do well, students needed to spontaneously use the four categories of website evaluation and the 14 questions within those categories that they were taught during the lessons.

After the final test was completed and student data were finalized, students were matched across the pre-test, teaching sessions, and the final test, in order to make sure that each student who had their data analyzed was present on all days of the lessons. Any student who missed a day of the lesson was removed from the data analysis.

## Analyses

For our primary analyses, a three-level hierarchical linear mixed-effects model (HLM) was used, with students (level 1) nested within classroom (level 2), which in turn was nested within schools (level 3). Of specific interest was the relationship between student (level 1) and ISI (level 1 predictor variable) on the different dependent variables (the final test measures).

We followed the HLM with Bayesian t-tests and one-way ANOVAs to verify null and indeterminate claims. We used default priors (Rouder et al., 2012). Before running the analyses, tests were conducted to ensure that assumptions were satisfied for the ANOVAs. There were violations of normality in every sample. A nonparametric test (Mann–Whitney U) was run on the ranks when possible to ensure the accuracy of the results. These results showed the same outcome as the t-tests on the final test measures. Levene’s test for equality of variance was conducted for each test under the requirement of *p* > 0.05. When this assumption was violated, degrees of freedom were adjusted.

Due to the inclusive nature of the study, no outliers were removed. However, efforts were made to check (post-hoc) if the results would differ when certain groups of students were removed from the analyses: (1) students with self-reported effort scores of 0 or 1 out of a possible 5 at final test (*n* = 42); (2) students with self-reported effort scores of 0 or 1 out of a possible 5 during the learning sessions *and* at final test (lesson 1, *n* = 16; lesson 2, *n* = 18; lesson 3, *n* = 23; final test, *n* = 42); (3) students who had any missing data during the learning sessions, since we could not be sure that they were completing the full task (*n* = 46), and (4) one class in the spaced condition that scored significantly lower marks at final test (*n* = 19). Removal of data was attempted one at a time and in combination and showed no difference in results. Therefore, the following results include our entire final sample of *n* = 716. Table 6 contains a summary of the HLM data, and Table 7 contains a full summary of the ANOVA data.

**Table 6:** Summary of HLM data

Predictors				Website 1						Website 2					
	Categories recalled /4			Categories used /14			Questions used /4			Categories used /14			Questions used /4		
	Estimates	CI	*p*	Estimates	CI	*p*	Estimates	CI	*p*	Estimates	CI	*p*	Estimates	CI	*p*
Intercept	2.16	1.86, 2.46	< 0.001	2.20	1.91, 2.49	< 0.001	2.03	1.68, 2.39	< 0.001	4.09	3.35, 4.82	< 0.001	3.25	2.58, 3.91	< 0.001
ISI [spaced]	0.31	− 0.01, 0.62	0.057	− 0.07	− 0.42, 0.28	0.71	− 0.03	− 0.43, 0.37	0.88	0.09	− 0.77, 0.94	0.84	− 0.02	− 0.78, 0.74	0.69

Accuracy scores for categories and questions in the spaced and massed conditions, at pre-test and at final test

**Table 7:** Summary of data

	Massed				Spaced			
	*n*	*M*	SD	95% CI	*n*	*M*	SD	95% CI
**Pre-test paragraph**								
Categories used (/4)	367	0.85	0.697	0.78, 0.92	349	0.94	0.723	0.87, 1.02
Questions used (/14)	367	1.43	1.21	1.30, 1.55	349	1.33	1.19	1.20, 1.46
**Final test paragraph**								
Website 1								
Categories used (/4)	365	2.3	1.03	2.19, 2.40	348	2.2	1.11	2.09, 2.33
Questions used (/14)	365	4.44	2.5	4.18, 4.69	348	4.43	2.67	4.06, 4.62
Website 2								
Categories used (/4)	363	2.17	1.16	2.05, 2.28	340	2.1	1.22	1.97, 2.23
Questions used (/14)	363	3.44	2.23	3.22, 3.68	340	3.4	2.33	3.15, 3.65
**Final test recall**								
Categories recalled (/4)	367	2.3	1.4	2.16, 2.45	349	2.58	1.3	2.45, 2.73
Website ratings*								
Pre-test	367	2.34	1.25	2.20, 2.48	349	2.26	1.3	2.12, 2.41
Lesson 1	346	2.23	1.4	2.06, 2.36	324	2.27	1.35	2.13, 2.44
Lesson 2	349	2.84	1.98	2.64, 3.07	331	2.31	1.82	2.12, 2.54
Lesson 3	352	1.68	1.36	1.48, 1.77	341	2	1.82	1.81, 2.23
Final test 1	367	1.99	1.64	1.84, 2.21	349	2.04	1.66	1.83, 2.20
Final test 2	363	2.7	2.1	2.39, 2.85	341	2.66	1.89	2.38, 2.80

Accuracy scores for categories and questions in the spaced and massed conditions, at pre-test and at final test

*Website ratings are based on difference scores (absolute value of student rating − correct rating)

## Results

### Baseline

Use of the four categories and 14 questions in the pre-test rating paragraph was evaluated, in order to ensure that students did not already know the material and as a check on the sufficiency of random assignment and stratification procedures. Since all responses were marked by hand and paraphrasing was accepted (e.g., “who made the website” earned a mark in the author category), two blind raters marked student responses (see “Appendix” for typical examples of student response paragraphs for each website). Students were marked out of four on which categories they mentioned in the paragraph, and out of 14 on which specific questions they chose and/or remembered to use in their rating explanation. Inter-rater reliability calculated from Pearson’s *r* was 0.71 (website 1) and 0.82 (website 2) for the four categories and 0.81 (website 1) and 0.88 (website 2) for the 14 questions. The final marks were determined by taking an average between the two raters. Massed and spaced groups did not differ on how many of the four categories were used to explain their rating in a paragraph, *d* = -0.12, BF_10_ = 0.31. Groups also did not differ in their use of the 14 questions in a paragraph, *d* = 0.08, BF_10_ = 0.15, or on the initial ratings, *d* = 0.07, BF_10_ = 0.15. Bayes factors suggested substantial evidence that the groups had equal performance at baseline (Jarosz & Wiley, 2014). Therefore, we proceeded with our analyses as planned.

### Learning of critical thinking skills

We examined whether students’ use of categories and questions increased between the pre-test and the final test, as a check that the lessons improved their critical thinking skills. This was true for all four pairwise ANOVAs comparing pre-test and final test question and category scores (pre-test vs. final website 1 categories: *d* = 1.2, BF_10_ = ∞; pre-test vs. final website 2 categories: *d* = 1.0, BF_10_ = ∞; pre-test vs. final website 1 questions: *d* = 1.2, BF_10_ = ∞; pre-test vs. final website 2 questions: *d* = 0.91, BF_10_ = ∞).

### Hypothesis 1: fact learning tested via recall

We predicted that students in the spacing condition, when asked directly what the four categories were (design, authority, content, and purpose), would recall more from the lessons than students in the massed condition at final test. Students in the massed condition remembered an average of 2.3 out of the four categories, and students in the spaced condition remembered an average of 2.58 categories. In the previous study by Foot-Seymour et al. (2019), students in the massed condition only remembered an average of 1.2 categories—a much lower average than the current value. Although Bayesian analyses showed that students in the spaced condition had higher category recall (*d* = 0.21; BF_10_ = 4.02), results of the HLM were not significant, *β* = 0.31, 95% CI [-0.01, 0.62], *p* = 0.57.

### Hypothesis 2: critical thinking tested via open-ended paragraph

We predicted that the spacing effect would benefit critical thinking: Students in the spaced condition will spontaneously use more information in an open-ended paragraph, by giving details taught in the lessons to explain their website ratings, than students in the massed condition. This was prompted by giving students two different websites at final test and simply asking for each, “Is this website credible? Please explain using evidence from the website.” All our analyses supported the null hypothesis: Students in the spaced condition did not use more of the four categories (*M* = 2.20 for website 1 and *M* = 2.10 for website 2) to explain their rating than students in the massed condition (*M* = 2.20 for website 1 and *M* = 2.17 for website 2) for website 1 (*β* = -0.07, 95% CI [-0.42, 0.28], *p* = 0.71; *d* = 0.09; BF_10_ = 0.17) or website 2 (*β* = -0.03, 95% CI [-0.43, 0.37], *p* = 0.877; *d* = 0.06; BF_10_ = 0.11). Students in the spaced condition did not use more of the 14 questions (*M* = 4.43 for website 1 and *M* = 3.40 for website 2) to explain their rating than students in the massed condition (*M* = 4.44 for website 1 and *M* = 3.44 for website 2) for website 1 (*β* = 0.09, 95% CI [-0.77, 0.94], *p* = 0.84.; *d* = 0.05; BF_10_ = 0.10) or website 2 (*β* = -0.02, 95% CI [-0.78, 0.74], *p* = 0.96.; *d* = 0.02; *BF*_10_ = 0.086). This contradicts the results of the previous study by Foot-Seymour et al. (2019), where there was an effect of critical thinking in the paragraph for the one website that was evaluated—students in the massed condition used an average of 0.7 categories and 3.2 questions in the paragraph, and students in the spaced condition used an average of 1.2 categories and 4.1 questions in the paragraph.

### Hypothesis 3: ratings during lessons

We predicted that students who were in the massed condition would be better at rating the websites during the daily lessons than students in the spaced condition, during the weekly lessons. This is due to the previously explained phenomenon that massed learning is more efficient than spaced learning in the moment*,* benefiting short-term retention but sacrificing it in the long term. Our results indicated that there was no difference in website rating accuracy after learning occurred in lesson one, *d* = 0.03, BF_10_ = 0.09. The spaced group performed better during lesson two than the massed group, *d* = 0.29, BF_10_ = 53.23. The massed group was closer to the correct rating after lesson three, although evidence was indeterminate*, d* = 0.20, BF_10_ = 2.27.

### Hypothesis 4: website ratings at final test

At final test (35 days later), we predicted that students in the spaced condition would rate both websites more accurately than students in the massed condition. Our analyses indicated that there were no differences in ratings between the spaced and massed groups on the final test websites, for either website 1, *d* = 0.004, BF_10_ = 0.09, or website 2, *d* = 0.014, BF_10_ = 0.09.

### Knowledge recall versus use of critical thinking skills

We examined whether students recalled more categories than they used in website evaluation paragraphs, at final test. We ran mixed ANOVAs, with test type (recall or usage) as a within-subjects factor, and inter-study interval as a between-subjects factor. Students recalled more questions than they used, for website 1, η^2^ = 0.006, BF_10_ = 66.3, and website 2, η^2^ = 0.015, BF_10_ = 370,584. There was an interaction between test type and inter-study interval for website 1, η^2^ = 0.006, BF_10_ = 78.8, and website 2, η^2^ = 0.005, BF_10_ = 16.5. Paired sample t-tests showed that students in the spaced condition recalled more categories than they used (website 1: *d* = 0.30, BF_10_ = 207,505; website 2: *d* = 0.34, BF_10_ = 7,845,000). This was not true for students in the massed condition (website 1: *d* = 0.006, BF_10_ = 0.059; website 2: *d* = 0.095, BF_10_ = 0.30).

## Discussion

The main goal of this study was to see whether the robust spacing effects seen in the laboratory could also be seen in the classroom under real-world conditions, using curriculum-based materials involving critical thinking. Additionally, this study investigated whether the results from the Foot-Seymour et al. (2019) study could be replicated in the classroom with real teachers leading the intervention. There were both expected and unexpected findings, which was not surprising since effectiveness trials by nature are intended to account for all of the external factors that could potentially decrease an intervention’s effect and lessen its effect size. Like decades of laboratory-based studies, we found a spacing effect for category recall. An effect size of *d* = 0.21 was seen in category learning compared to an effect size of *d* = 0.85 for a similar measure in our closely related efficacy trial (Foot-Seymour et al., 2019), which was likely a side effect of the release of control and added noise. Other classroom studies are somewhere in between, with a mean effect size for fact learning of *d* = 0.47 (Carpenter et al., 2009; Kapler et al., 2015).

Although traditional spacing studies only contain two study sessions, we added a third session so that students could have another opportunity to practice their website evaluations. This mimics standard teaching practice; likewise, researchers used three learning sessions in a closely related efficacy trial (Foot-Seymour et al., 2019). This decision had some repercussions—for example, students were removed from the study if they missed a lesson, and adding another session increased the likelihood that removal would happen.

Our lesson plans were designed so that by the end of all three lessons, students should have been able to effectively judge the credibility of websites. Indeed, our analyses demonstrated a large improvement in critical thinking skills as a result of the lessons, suggesting that effectiveness of credibility judgments greatly increased. Students were skeptical of the websites they saw and were able to use collected evidence via the website evaluation checklist to explain their credibility ratings.

We were surprised that during the final test, students in the spaced condition chose not to use some of the categories they knew, when explaining their ratings in a paragraph. Students in the massed condition used as many categories as they recalled, while writing rating justification paragraphs, demonstrating concordance between knowledge and usage. Students in the spaced condition used fewer categories than they knew about. We are not able to determine why they failed to use all the evaluation criteria they remembered when writing their rating justification paragraphs.

Students have been shown to enjoy an online learning environment more than traditional in-person teaching, since it can promote learning that can be less intimidating and encourage participation and meaningful interactions (Ni, 2013). This was qualitatively observed by teachers and volunteers during the current study—feedback from students that was collected after each lesson demonstrated that most enjoyed the online nature of the lessons. However, since there was no person on the other side of the screen and the majority of the information was given passively via YouTube videos, we cannot be sure that students were fully engaged and absorbed the material. This could have impacted the results of the study, since research has demonstrated that one of the biggest predictors of academic success is student engagement—particularly engagement with peers, the teacher, and the course material (Reiken et al., 2018). While some studies have shown no difference in learning between online and in-person lessons (Russel, 1999), other research has shown that success in an online course is very much dependent upon the nature of student to student and student to teacher interaction (Picciano, 2002). Therefore, it should not be automatically assumed that online and in-person teaching will result in the same learning outcomes (Manning-Oullette & Black, 2017).

### Challenges and limitations

The main challenge and limitation of this study was one that is present in all classroom studies—there was a lack of scientific control. Each class is composed of its own group of individuals who have different social, emotional, and academic needs. During recruitment, we requested that teachers let all students in the room participate if possible, which increased the variability of our sample, albeit in the way that we wanted since it was an effectiveness trial.

Although all teachers were certified by the Ontario College of Teachers, levels of experience varied (one mother-daughter duo, for example). Teachers have different teaching styles and different personalities and approaches. We tried to include a sufficient number of teachers in each condition so that mean teacher experience and effectiveness would be random. We also attempted to account for teacher variance using mixed-model analyses. These analyses supported our primary analyses, so it is unlikely that teacher differences are responsible for the pattern of results.

There are interruptions in a typical school day. During the 129 lessons that took place as part of this study, we had four snow days, fire drills, a power outage, and several interruptions of Internet service. There were several times when teachers had an interruption to their day and had to reschedule one or all of the lessons. In order to keep track of these interruptions when lessons were teacher-led, volunteers were sent to the classroom as much as possible. There were changes in context from class to class. Some teachers opted to run the lessons in their normal classroom, others in the computer laboratory or library, and each school varied in terms of space. At every stage, teachers were given the freedom to use their normal practice. All of these challenges were expected, and we prepared by collecting a large sample size, hoping that the random assignment of classrooms would handle these differences.

Lesson plans and online materials were released to teachers with minimal additional instruction with the expectation that they would carry out the intervention as planned. Lesson plans were created to follow typical practice and could have been followed easily by any certified teacher. Most of the time, participating teachers did what they were asked to do and took interest in the research. Other times, it was clear that they had originally agreed because they saw value in the research topic but had not read the lesson plans in advance and therefore did not remember that they were responsible for some of the teaching. In order to manage this challenge and add value to the lessons, the lead researcher ran the lesson on day one to model the day for teachers and show them that since the majority of the lessons were online, there was minimal in-person teaching required.

Teacher preparedness may have affected the results of the lessons, and therefore the final test. The results of the final test depended on students’ use of all the questions and categories in the paragraphs throughout the whole process. It was important for students to know that they needed to use as many questions and categories as possible. In Foot-Seymour et al. (2019), researchers were always present and were consistently reinforcing the use of these in the paragraphs. However, it is possible that if teachers didn’t read the lesson plans closely and prepare for lesson delivery, they may not have been continually reinforcing the same things. As a result, students may not have chosen to respond with as many questions and categories as they remembered, so if they deemed certain categories more important than others, they may have chosen to focus their attention on those categories and not on others.

There was a difference not only in lesson preparedness but also in teacher enthusiasm for the lessons. Enthusiasm carried across from teachers to their students—classes where teachers were keen and prepared seemed to be more engaged. Students from these classes were more likely to write in comments about how much they enjoyed the lessons. This was a natural side effect of the study, since effectiveness trials are intended to see what happens under normal conditions, which includes differences in teacher engagement.

Teachers needed to know up front what the procedure was for teaching the lessons, so they could not be blinded to conditions; they knew whether they were in the massed or spaced condition. We could not blind them to the hypotheses, a requirement of the external research board for York Region. However, it was not concerning that teachers were not blinded to the main hypotheses, since each was responsible for their own classroom and it was unlikely that they would alter their teaching practice to cause any bias.

Lastly, as highlighted in the results section, the massed group scored significantly higher on average on the category recall measure than in the previous study by Foot-Seymour et al. (2019). This could have been due to any extraneous factors that were beyond researcher control. When students were asked to recall the four categories of website evaluation, students in the previous study remembered an average of 1.2 (massed) and 2.3 categories (spaced). In the current study, students ranged on average from 2.1 to 2.2 categories, values which are much closer to the spacing group in the previous study. This difference could indicate any number of things. Students might have all learned the materials better than in the previous study (for reasons unknown), there could have been some cheating on final test (since the researchers were absent), the change in age group might have impacted results, or any number of other differences that separated previous studies from this one.

## Conclusion

Implementation of the curriculum is something that should be considered whenever planning long-term learning goals for the school year. If the goal is for students to retain as much information as possible, teachers need to be aware of cognitive strategies like the spacing effect, so that they can make small changes to their teaching practice to help students become more successful. A possible barrier to this might be that teaching resources are cumulative, and teachers often use similar materials from year to year, so asking them to change their plans entirely could be intrusive and intimidating. The benefit of using spacing is that the only adjustment that needs to be made is in the timing of long-range plans. This not only is achievable but also would be beneficial to both students and teachers by saving time in the long run.

As a next step, researchers should run effectiveness trials with different subject materials and a wide range of measurements. Critical thinking was a major focus in the current study, which is why website evaluation was used. This choice served to enhance our recruitment because it added value to teaching programs that were already in place. By using additional types of curriculum-based subject material, the results that we saw during the fact learning measures can be more fully evaluated and used to decide if spacing should be recommended in a real-world classroom setting. Overall evidence to date suggests that spacing lessons can benefit fact learning in real-world classrooms, and there is likely a benefit to critical thinking skills as well.

### Supplementary Information

**Additional file 1.** Curriculum Materials and Teacher Information.

## Data Availability

The dataset and materials supporting the conclusions of this article are available in the OSF repository, https://osf.io/9zjt2/.

## Appendix

Typical examples of student paragraphs for each website used during the lessons.

**Table:**

Sea Monkey Online (Student Rating: 8/10)	
Firstly, the site has incorrect spelling on the name of the University, on the site, it is spelled "MacGill University" and not McGill University. Additionally, in the resources section, all the websites are listed (not cited in APA format), and under the website is an arbitrary picture of a shrimp that looks out of place. In addition, the pictures do not look professional, considering the fact that in one that there is an infant in one image holding a used package of "Sea Monkeys". On the site, the author is not recognized, only when you click on the "The Office of Science and Society" is he recognized. Everything looks relevant and all the information looks correct, however, there is one unprofessional video, made by what seems to be a video producer that does not seem to inform the audience, and is made for entertainment purposes only. Although the website itself is not trying to sell the product to the audience, there are multiple links to purchase them	
Brain Science (Student Rating: 0/10)	
The authority of this website is terrible. When you search up the address of Brain Science Inc. all you see is a deserted area with something that looks like a hotel nearby. The author isn't mentioned, except for the beginning part in which they say a Dr. Daniel Reid wrote the first page. They claim he is an American neuroscientist, but he is actually a physician from Nova Scotia. I doubt he wrote that first page. The website's content is even worse than its authority. Almost every fact written on it isn't true, save for the first page where only the most elementary elements of the workings of the brain are listed. Otherwise the site is a mess as far as content goes. The website barely even talks about the science of the brain, and instead rants on and on about some miracle pill that is clearly just a placebo. The brain information that is there is for the must [sic] part inaccurate. It's been proven that we don't use only 10% of our brain, and we don't need a vitamin to make it work right. The testimonials are also fake. John Green doesn't even live in Ohio! Plus, some of the ingredients in Neuroflex are not even real things. Overall, I think the most important thing about any site is it's [sic] info. The information in this site was not true at all, which is why I rated it at a 0. Although the design could be rated at a 3 or 4, everything else is a 1 or 0. I would not trust this website at all	
Bizarre Animals (Student Rating: 4/10)	
The design was a little childish, not very professional. But maybe it was meant for kids. It was most likely for educational purposes because the author isn't trying to sell anything, and there is good educational information on it. There were links that took you to websites that are irrelevant to the website's purpose and topic, so that was a red flag for me. I was confused. It says that the author is "staff," but I don't know who that is. There is no way of contacting the author so that is a huge red flag for me. Also, in one of the videos, it shows a gulper eel stretching its jaw. It looked very, very fake. It looked photoshopped. But I don't know for sure. So overall, I think it was for educational purposes, but there were a couple red flags for me. It contained some great information, but the website itself was a bit unprofessional. I would rate it higher because of the information, but lower for its unprofessionalism	
Glow-in-the-Dark Bunnies (Student Rating: 6/10)	
The design of the website is a bit hard to read as there isn't any headings but there small is subheadings. The background of the page is a bright neon green and the text is a light green this makes the text a bit hard to read and could impair understanding or comprehension. The author is stated at the top and doing a little bit of research on her I found out that she is the editor of the MIT technology review and is somewhat of an expert in the subject which makes the information valid. The purpose of the page is to educate you and doesn't try to sell you anything. In most of the subheading if there is any sort of research done the source is cited and is given credit for	
Association of Geniuses (Student Rating: 3/10)	
The design of the website is very professional, as the fonts, colour schemes and format are all neat, clear, and organised. The authority of the website is truly dreadful. There is no clear identification of the author, and the photos of members are just random stock images of children, with no identification. The only method of communication is an obviously anonymous email, but other than that, pretty much nothing is well [sic] in terms of credibility. The content is truly a sight to forget. The information about Eisenstein's childhood and equations are wrong. The website stated that *E* = *MC*^2^ is the gravity equation. BUT, it is the relativity equation, relating matter and energy as 2 forms of the same thing! NOT GRAVITY! Along with that, the website stated that Einstein was bad at math in his childhood, and liked to draw. This is far from the truth, as Einstein loved math and science, he just was very inquisitive in the classroom. The only redeeming quality of the content is that the links and websites are actually realistic and truthful, but is that really good enough to fix up the twisted lies this website makes up? Finally, we have our purpose. The purpose is to get the reader to donate to help struggling students. To do this, they make up lies and unrealistic information to make it look like he was a struggling student, so you could help other struggling students. The website used the purpose of money and funding to lie, and convince the reader that these students are the next Einsteins, but really, they just want your money	

## Abbreviations

NCTQ: National Council on Teacher Quality
ISI: Inter-study interval
RI: Retention interval

## References

[CR1] Abrami PC, Bernard RM, Borokhovski E, Wade A, Surkes M, Tamim R, Zhang DA (2008). Instructional interventions affecting critical thinking skills and dispositions: A stage 1 meta-analysis. Review of Educational Research.

[CR2] Angeli C, Valanides N (2009). Instructional effects on critical thinking: Performance on ill-defined issues. Learning and Instruction.

[CR3] Bjork EL, Bjork RA, Gernsbacher MA, Pew RW, Hough LM, Pomerantz JR (2011). Making things hard on yourself, but in a good way: Creating desirable difficulties to enhance learning. Psychology and the real world: Essays illustrating fundamental contributions to society.

[CR302] Bloom BS (1956). Taxonomy of educational objectives.

[CR4] Bloom KC, Shuell TJ (1981). Effects of massed and distributed practice on the learning and retention of second-language vocabulary. Journal of Educational Research.

[CR5] Bronstein, D. M. (2007). *The efficacy of a web site evaluation checklist as a pedagogical approach for teaching students to critically evaluate internet content* (Unpublished doctoral dissertation). The Graduate School of Computer and Information Sciences Nova Southeastern University.

[CR6] Butler AC, Marsh EJ, Slavinsky JP, Baraniuk RG (2014). Integrating cognitive science and technology improves learning in a STEM Classroom. Educational Psychology Review.

[CR7] Carpenter SK, Pashler H, Cepeda NJ (2009). Using tests to enhance 8th grade students’ retention of U.S. history facts. Applied Cognitive Psychology.

[CR8] Cepeda NJ, Coburn N, Rohrer D, Wixted JT, Mozer MC, Pashler H (2009). Optimizing distributed practice: Theoretical analysis and practical implications. Experimental Psychology.

[CR9] Cepeda NJ, Pashler H, Vul E, Wixted JT, Rohrer D (2006). Distributed practice in verbal recall tasks: A review and quantitative synthesis. Psychological Bulletin.

[CR10] Cepeda NJ, Vul E, Rohrer D, Wixted JT, Pashler H (2008). Spacing effects in learning: A temporal ridgeline of optimal retention. Psychological Science.

[CR11] Delaney PF, Verkoeijen PP, Spirgel A (2010). Spacing and testing effects: A deeply critical, lengthy, and at times discursive review of the literature. Psychology of Learning and Motivation.

[CR12] Dempster FN, Bjork EL, Bjork RL (1996). Distributing and managing the conditions of encoding and practice. Memory.

[CR13] DeRemer P, D’Agostino PR (1974). Locus of distributed lag effect in free recall. Journal of Verbal Learning and Verbal Behavior.

[CR14] Dewey J (1909). How we think.

[CR15] Ennis RH, Baron JB, Sternberg RJ (1987). A taxonomy of critical thinking dispositions and abilities. Teaching thinking skills: Theory and practice.

[CR16] Ennis RH (2018). Critical thinking across the curriculum: A vision. Topoi.

[CR17] Facione PA (1990). Critical thinking: A statement of expert consensus for purposes of educational assessment and instruction. Research findings and recommendations.

[CR18] Foot-Seymour V, Foot J, Wiseheart M (2019). Judging credibility: Can spaced lessons help students think more critically online?. Applied Cognitive Psychology.

[CR19] Fullan, M. (2013). *Great to excellent: Launching the next stage of Ontario’s education agenda*. Ontario Ministry of Education. http://www.edu.gov.on.ca/eng/document/reports/FullanReport_EN_07.pdf

[CR21] Gluckman M, Vlach HA, Sandhofer CM (2014). Spacing simultaneously promotes multiple forms of learning in children's science curriculum. Applied Cognitive Psychology.

[CR22] Greenberg, J., Pomerance, L., & Walsh, K. (2016). *Learning about learning: What every new teacher needs to know*. National Council on Teacher Quality. http://www.nctq.org/dmsView/Learning_About_Learning_Report

[CR23] Harden RM (1999). What is a spiral curriculum?. Medical Teacher.

[CR304] Jarosz AF, Wiley J (2014). What are the odds? A practical guide to computing and reporting Bayes factors. Journal of Problem Solving.

[CR24] Janiszewski C, Noel H, Sawyer AG (2003). A meta-analysis of the spacing effect in verbal learning: Implications for research on advertising repetition and consumer memory. Journal of Consumer Research.

[CR25] Kapler IV, Weston T, Wiseheart M (2015). Long-term retention benefits from the spacing effect in a simulated undergraduate classroom using simple and complex curriculum material. Learning and Instruction.

[CR26] Küpper-Tetzel CE, Erdfelder E, Dickhauser O (2014). The lag effect in secondary school classrooms: Enhancing students' memory for vocabulary. Instructional Science.

[CR27] Kuhn D (1999). A developmental model of critical thinking. Educational Researcher.

[CR300] Maddox GB (2016). Understanding the underlying mechanism of the spacing effect in verbal learning: A case for encoding variability and study-phase retrieval. Journal of Cognitive Psychology.

[CR28] Manning-Oullette A, Black KM (2017). Learning leadership: A qualitative study on the differences of student learning in online versus traditional courses in a leadership studies program. Journal of Leadership Education.

[CR29] Moss, V. D. (1995). *The efficacy of massed versus distributed practice as a function of desired learning outcomes and grade level of the student* (Publication no. 9603493) [Doctoral dissertation, Utah State University]. ProQuest Dissertations Publishing.

[CR30] Ni AY (2013). Comparing the effectiveness of classroom and online learning: Teaching research methods. Journal of Public Affairs Education.

[CR31] Ontario Ministry of Education. (2006). *The Ontario curriculum grades 1–8: Language*. http://www.edu.gov.on.ca/eng/curriculum/elementary/language18currb.pdf

[CR32] Picciano AG (2002). Beyond student perceptions: Issues of interaction, presence, and performance in an online course. Journal of Asynchronous Learning Networks.

[CR33] Rawson KA, Kintsch W (2005). Rereading effects depend on time of test. Journal of Educational Psychology.

[CR34] Rea CP, Modigliani V (1985). The effect of expanded versus massed practice on the retention of multiplication facts and spelling lists. Human Learning: Journal of Practical Research & Applications.

[CR35] Reder LM, Anderson JR (1982). Effects of spacing and embellishment on memory for the main points of a text. Memory & Cognition.

[CR36] Reed HB (1924). Distributed practice in addition. Journal of Educational Psychology.

[CR37] Regional Municipality of York. (2016). *2016 census profile*. Retrieved January 10, 2019, from https://www.york.ca/wps/portal/yorkhome/yorkregion/yr/statisticsanddata/censusanddemographicdata/censusanddemographicdatalist

[CR38] Reiken CJ, Dotson WH, Carter SL, Griffith AK (2018). An evaluation of interteaching in an asynchronous online graduate-level behavior analysis course. Teaching of Psychology.

[CR39] Rohrer D (2009). Research commentary: The effects of spacing and mixing practice problems. Journal for Research in Mathematics Education.

[CR40] Rohrer D, Taylor K (2007). The shuffling of mathematics problems improves learning. Instructional Science.

[CR41] Rouder JN, Morey RD, Speckman PL, Province JM (2012). Default Bayes factors for ANOVA designs. Journal of Mathematical Psychology.

[CR42] Rovee-Collier C, Evancio S, Earley LA (1995). The time window hypothesis: Spacing effects. Infant Behavior and Development.

[CR43] Russel TL (1999). The no significant difference phenomenon.

[CR44] Seabrook R, Brown GDA, Solity JE (2005). Distributed and massed practice: From laboratory to classroom. Applied Cognitive Psychology.

[CR45] Siegel H (1988). Educating reason: Rationality, critical thinking, and education.

[CR46] Simone PM, Bell MC, Cepeda NJ (2013). Diminished but not forgotten: Effects of aging on magnitude of spacing effect benefits. The Journals of Gerontology Series b: Psychological Sciences and Social Sciences.

[CR47] Sobel HS, Cepeda NJ, Kapler IV (2011). Spacing effects in real-world classroom vocabulary learning. Applied Cognitive Psychology.

[CR48] Taraban R, Rynearson K, Stalcup KA (2001). Time as a variable in learning on the world-wide web. Behavior Research Methods, Instruments, & Computers.

[CR49] Toppino TC, Gerbier E, Ross BH (2014). About practice: Repetition, spacing, and abstraction. Psychology of learning and motivation.

[CR50] Verkoeijen PPJL, Rikers RMJP, Özsoy B (2008). Distributed rereading can hurt the spacing effect in text memory. Applied Cognitive Psychology.

[CR51] Vlach HA, Sandhofer CM (2012). Distributing learning over time: The spacing effect in children’s acquisition and generalization of science concepts. Child Development.

[CR52] Wiseheart M, Küpper-Tezel C, Weston T, Kim ASN, Kapler IV, Foot-Seymour V, Dunlosky J, Rawson K (2019). Enhancing the quality of student learning using distributed practice. Cambridge handbook of cognition and education.

[CR53] Zechmeister EB, Shaughnessy JJ (1980). When you know that you know and when you think that you know but you don't. Bulletin of the Psychonomic Society.

