# Supplementary material for: Judging the credibility of websites: an effectiveness trial of the spacing effect in the elementary classroom
Source: Cogn Res Princ Implic. 2022 Jan 17;7:5. doi: 10.1186/s41235-022-00358-w (PMC8763985; doi:10.1186/s41235-022-00358-w)
Supplement: Supplementary file 1 — Additional file 1. Curriculum Materials and Teacher Information. [file 41235_2022_358_MOESM1_ESM.pdf]

To Our Wonderful Participating Teachers,

I hope that you and your students enjoy these lessons! They have been designed for easy implementation—the majority of the teaching materials are online. Each student will need his or her own computer and a pair of headphones. You can use your own room or take your students to the library or computer lab.

Throughout these online lessons, your students will be learning to judge the credibility of websites. They will become skeptical of the websites they see and will learn to use collected evidence via the online website evaluation checklist (a.k.a. the "scavenger hunt") to explain their credibility ratings. The evidence that they collect will be used to support a final rating of 0-10 (0-4 is not credible; 5 is neutral; 6-10 is credible), and while rating accuracy will improve throughout the lessons, the most important aspect is the *process* that leads them to their final decision. By *searching* for the author, *questioning* the site's purpose, *exploring* the content and *evaluating* the site's design, your students are building skills which will make them better critical consumers of the internet.

Your job is to facilitate the delivery of these lessons within the timeframe discussed: Each lesson will require one language block (3 x approximately 90 minutes), followed-up by an additional block one-month later. The reason that we have set the timing for you, is because spacing out learning has been shown time and time again to be effective for retention. For the first time, we get to work together to see if it works with all of the unpredictability that comes with a "regular" classroom. As long as you teach the lessons in the timing that we discussed and focus on the learning objectives, you can do everything else that you would normally do otherwise. That being said, here are a few specific scientific controls that we DO need to have: Please don't reveal our hypothesis re: spacing to students and please do not formally review the lesson's categories or questions within 30 days of the last lesson—it may be impossible to avoid having students talk about it, but please don't encourage these potential discussions. These controls are necessary so that we can properly assess their memory and I appreciate your assistance in this regard. Once the lessons are done, if you need the information for your own assessment purposes, you can ask your principal to e-mail me and I will send you all of the data. Otherwise, I'll give you a separate assessment once the lessons are completely finished.

If you know of any other teachers who may want to participate, please ask them to contact me. The more, the merrier! Once all of the lessons are done, I will make the materials open access so that you and your colleagues can use them in the future.

Thank you very much for participating! Please read the lesson plans carefully and review all of the materials before running the lessons. If you have any questions, please check the FAQ on the last page and/or feel free to contact me.

JUDGING THE CREDIBILITY OF ONLINE SOURCES: OVERVIEW

LEARNING OBJECTIVE(S)

By the end of these lessons, students will be able to effectively judge the credibility of websites. They will be skeptical of the websites they see and will be able to use collected evidence via the website evaluation checklist (a.k.a. the "scavenger hunt") to *explain* their credibility ratings. The evidence that they collect will be used to support a final rating of 0-10 (0-4 is not credible; 5 is neutral; 6-10 is credible), and while rating accuracy will improve throughout the lessons, they will learn that the most important aspect is the *process* that leads them to their final decision.

MATERIALS

Computers with internet access, headphones, markers, chart paper, teacher projector & computer

	WEBSITE(S)	TASKS
BEFORE		*Teachers, please review lesson plans and materials and ensure all participating students have parental consent.*
DAY ONE	**Sea Monkey**<br><br>www.seamonkeyonline.wordpress.com	Introduction (In Class)<br><br>Lesson One (Online)<br><br>Discussion (In Class)
DAY TWO	**Brain Science**<br><br>www.brainsciencenow.wordpress.com	Review (In Class)<br><br>Lesson Two (Online)<br><br>Discussion (In Class)
DAY THREE	**Bizarre Animals**<br><br>www.bizarreanimalworld.wordpress.com	Review (In Class)<br><br>Lesson Three (Online)<br><br>Discussion (In Class)<br><br>*Teachers, for the next month, please do not do any refresher activities.*
FINAL TEST<br><br>(ONE MONTH LATER)<br><br>WILL CONTACT	**Research Science**<br><br>www.researchsciencetoday.wordpress.com<br><br>**Association of Geniuses**<br><br>www.associationofgeniuses.wordpress.com	Final Test (Online)<br><br>Discussion (In Class)

Lesson One

Time Required	Website	Tasks	Materials
Literacy block (90-100 mins)	**Sea Monkey**<br>www.seamonkeyonline.wordpress.com	1. Introduction (In Class)<br>2. Lesson One (Online)<br>3. Discussion (In Class)	• Computers with internet access and headphones for each student<br>• Teacher projector and computer<br>• Chart paper and markers

1. **Introduction (5 minutes)**

Tell students that they'll be learning to judge the credibility of websites. These lessons have been designed for them and are entirely online! Students will log in to their computers. Write this URL on the board for students to copy into their browser *(www.credibilitylesson1.weebly.com).* You may need to explain to the students what a URL is and remind them of how to type it in without doing a Google search. The URL will contain the link to the lessons and the link to the website if they close it by mistake. You can ask students to help a buddy if they are having trouble typing in the URL. The survey auto-saves so they will never lose work if they close it or need to go back.

2. **Lesson One: Online (60 minutes)**

Students do the lesson <u>on their own computer</u>. The lesson will lead students through a diagnostic assessment (assessment *for* learning). Students will be asked not to talk during this task. This should be challenging, and students can write things like, "I don't know how to do this" if they are struggling.

Then, students continue to learn about the four categories of website design: design, authority, content and purpose, and how they can be used to identify whether a website is credible or not. They will be taught how to use the scavenger hunt to dive deeper into the website. **Students can whisper if they want to but should be encouraged to write down everything that is exciting enough to share.**

Most of this lesson should run on its' own. Please just circulate and help with technological assistance. Stick to your standard practice— do what you would normally do! As students finish the lesson, ask them to continue exploring the website. Alternatively, you can ask students to get a book or do other unfinished work (please don't give them a game or other online preferred activity to prevent students from rushing to finish).

3. **Discussion**

Log off of the computers and come together as a class. Say this to students: *when you first encountered this website, you were probably in a neutral position (50/50). You've never seen it before, so you probably didn't know what to think. However, as you explored the website, you saw things (maybe some red flags) that pushed you towards thinking it was credible or not credible. What were some things that you found?*

Choose some students to come to the front of the class to share collected evidence with everyone on the projector. Ask them to explain why the evidence pushed them towards thinking it was credible or not. Make a T-Chart on the board or on chart paper to keep track of the responses (one side will say "CREDIBLE", and the other side will say "NOT CREDIBLE"). Encourage them to focus on things they learned during the lesson.

Teacher Note: You don't need to keep the chart paper after. Please don't post it in the room for review. If you remember, please take a picture and text/email it to me!

Lesson Two

Time Required	Website	Tasks	Materials
Literacy block (90-100 mins)	**Brain Science**<br><br>www.brainsciencenow.wordpress.com	1. Review (In Class)<br>2. Lesson Two (Online)<br>3. Discussion (In Class)	• Computers with internet access and headphones for each student<br>• Teacher projector and computer<br>• Chart paper and markers

1. **Introduction (15 minutes)**

   Ask students, *what does credibility mean?*
   Answer: how **believable** and **trustworthy** something is.

   Ask them how they can decide whether they can believe or trust a website? Brainstorm as a class. *The answer is:* the four categories (design, authority, content and purpose). Then, break students into groups of 4-6 and give each group a piece of chart paper. Ask them to write down the categories as headings and list as many specific questions as they can remember. The goal of this activity is to get students to struggle slightly to remember. This should strengthen their memory trace. You can go around and give clues if students are stuck! The answer key is attached. Encourage students to try to organize the questions in the appropriate categories, but since some overlap (for example, the links question was sorted under design, but it could also be a content question), it doesn't really matter.

   Teacher Note: This is a brainstorming session. You don't need to keep the chart paper after. Please don't post it in the room for review.

2. **Lesson Two: Online (45 minutes)**

   Students will log in to their computers. They will go to *www.credibilitylesson2.weebly.com* (please write this on the board), and the browser will contain everything they need for the day. You can ask students to help a buddy if they are having trouble typing in the URL. The survey auto-saves so they will never lose work if they close it or need to go back. **Students can whisper if they want to but should be encouraged to write down everything that is exciting enough to share.**

   Most of this lesson should run on its' own. Please just circulate and help with technological assistance. Stick to your standard practice— do what you would normally do! As students finish the lesson, ask them to continue exploring the website. Alternatively, you can ask students to get a book or do other unfinished work (please don't give them a game or other online preferred activity to prevent students from rushing to finish).

3. **Discussion**

   Log off of the computers and come together as a class. Say this to students: *when you first encountered this website, you were probably in a neutral position (50/50). You've never seen it before, so you probably didn't know what to think. However, as you explored the website, you saw things (maybe some red flags) that pushed you towards thinking it was credible or not credible. What were some things that you found?*

   Choose some students to come to the front of the class to share collected evidence with everyone on the projector. Ask them to explain why the evidence pushed them towards thinking it was credible or not. Make a T-Chart on the board or on chart paper to keep track of the responses (one side will say "CREDIBLE", and the other side will say "NOT CREDIBLE"). Encourage them to focus on things they learned during the lesson

Lesson Three

Time Required	Website	Tasks	Materials
Literacy block (90-100 mins)	**Bizarre Animals**<br><br>www.bizarreanimalworld.wordpress.com	1. Review (In Class)<br>2. Lesson Two (Online)<br>3. Discussion (In Class)	• Computers with internet access and headphones for each student<br>• Teacher projector and computer<br>• Chart paper and markers

1. **Introduction (15 minutes)**

   Repeat introduction from Lesson Two.

2. **Lesson Three: Online (45 minutes)**

   This lesson is the same as Lesson Two, except that students will go to *www.credibilitylesson3.weebly.com*.

3. **Discussion**

   Repeat discussion from Lesson Two.

Final Test (Approximately One Month After Lesson 3: Will Contact with Reminder)

Time Required	Websites	Tasks	Materials
Literacy block (90-100 mins)	**Research Science**<br>www.researchsciencetoday.wordpress.com<br>**Association of Geniuses**<br>www.associationofgeniuses.wordpress.com	1. Final Test (Online)<br>2. Discussion (In Class)	• Computers with internet access and headphones for each student

1. **Introduction (2 minutes)**

   Tell students that they are going to be doing a website credibility test and evaluating two websites. Tell them to remember *as best as they can*. There is no pressure: if they don't remember something, they can leave it blank. Once they submit the final test, they may not come back to it later.

   This will be where we see whether the spaced or massed group remembered more from the lessons. We expect the average recalled categories to be around 2/4, and the average specific questions to be approximately 5/17. Please do not provide any hints and treat this like a normal test.

2. **Final Test: Online (60 minutes)**

   Students will log in to their computers. They will go to *www.credibilityfinaltest.weebly.com* (please write this on the board), and the browser will contain everything they need for the day. Students cannot chat during this final test.

   Just like the previous lessons, most of this lesson should run on its' own. Please just circulate and help with technological assistance. Stick to your standard practice here—do what you would normally do! As students finish the lesson, ask them to continue exploring the websites. If they find anything new, this time they **cannot** go back. Alternatively, you can ask students to get a book or do other unfinished work (please don't give them a game or other online preferred activity to prevent students from rushing to finish).

3. **Discussion: In Class**

   Log off of the computers and come together as a class. Ask students what they learned from the lessons. Have a group discussion about the final websites and reveal the truth about the final test websites. If there is extra time, students can share what they came across on the final two sites.

Frequently Asked Questions

What if students do not get consent to participate? Even though these lessons reflect standard teaching practice, it is still a research study, so we need parental consent for participation. If a student does not get permission to participate, please confirm whether or not they can still be physically present for the lessons. Most of the time, parents will let them stay in the room. In the case that parents do **not** let them stay in the room (this is unlikely), please make alternative arrangements for them.

What if I cannot run the lessons in the timing that we planned? Please try your best! I am trying to gather more evidence on whether lesson timing affects retention. If it does, together we might be able to make recommendations for professional development courses on the spacing effect. That being said, things happen!! If you are interrupted at any point (fire drill, snow day, internet goes down, etc.), follow up with the rest of the lesson ASAP and send me an e-mail to let me know. Try to keep the timing as close to what we planned as possible.

I have some kids who leave for language. Should they still go? You (and the SERT teacher) can make that decision. These lessons so that they can be used across a wide range of ages and capabilities. If your students stay, please let me know if there is any reason why I should expect different results from them compared to the rest of your class.

You are asking me to teach critical thinking. What is it? Our definition of critical thinking is simple. We are asking students to use reasonable, reflective doubt to decide what to believe or what to do. To be a good critical thinker, you have to be willing to deal with being unsure. You may notice that students have an issue with the uncertainties with the websites, but please keep encouraging them to just do their best at making a decision. The goal is not in deciding how credible they are—we care about the **quality** of their justification. The websites are purposefully ambiguous so that each student can use different reasoning to explain their perspective.

Can students use their iPhones or iPads? No. The screen is too small, and students need a keypad for the online survey. Please avoid using these devices.

What if a website is blocked, or if the technology doesn't work? I have tested all the URLs on the YRDSB wifi, but in the case that something happens, there is an "emergency kit" folder on the Google Drive. This has the PowerPoint, the videos, and offline checklists that you will be able to use. However, since this is a media study, if the students can't get on the websites (if the Internet is down), you won't be able to continue. Please handle the situation however you would normally (i.e., giving some DPA while you wait for the internet to come back up, moving around the daily schedule if possible).

Can I use any of this for my assessment? If you want the assessment that I collected for the research, please ask your principal to contact me and I'll release all of the data to them. However, once your class has completed all of the lessons, I will be sending a separate assessment for you to use.

Why are you talking about credibility and not having students identify real/fake sites? We have found that students want to use the terms "real/fake" because they're easier to conceptualize (it's very black and white!). However, we know in reality that judging website credibility is not so easy. There is a spectrum— website creators love to give opinions online, but that doesn't necessarily mean that the site itself is fake. At the end of the day, it's about how much we trust and believe what we encounter online. *Do you think that the travel blogger is an expert, and will you follow his/her to-do list when you go to Paris? What about the Pinterest recipe for angel food cake… do you think that it will work out?* We would rather have students be more skeptical than less skeptical. In a perfect world, students should target the red flags and assume everything is fake until being convinced otherwise. That's what these websites will do. Remember that whatever students decide, it's the <u>process of decision making</u> that matters.
